# Supplementary material for: Isolation and Functional Characterization of a Green-Tissue Promoter in Japonica Rice (Oryza sativa subsp. Japonica)
Source: Biology (Basel). 2022 Jul 22;11(8):1092. doi: 10.3390/biology11081092 (PMC9332004; doi:10.3390/biology11081092)
Supplement: Supplementary file 1 [file biology-11-01092-s001.zip › Supplementary information.pdf]

Os8GSX7 gene sequence (CDS)
ATGGCTAACATCAGAACCGTCTCCAGCCTCGGGGAGGTCAACGGTGCCCTGCAAGAG
ATGGGTATCAATACGATTGATCAAGCTCATCAAGTTCAATTTCGTTTACACAAGCAAA
CATCCCTTAAAGAAGCAACAGAAATAAAGATGATGATCCAAACTGGAAGGCATGGAT
TCAGGTTGGTTAACCCTGAGCTTTTAGATTGTAAGTTTGATGCCAGGGTTAAGCTAGA
GGAGTGGTACAATACCATGCTCGACGCATGCATGGCACAGTGTGATCATGAGCTGTT
CTCGCTTGAAGCTAGCATTGCGGAGCTAAAGGATCTCATGCTTTCCACAGACGATCAG
ATTCCACATATTGGACCTGAAGTTCACCACAGGAACCGTGGAGTGCAACAAATGCTC
TACCCAAACCCTCCATTCCCGATCGATCCTGATTATGAGTTTGGTACTCCGCAACAAC
GAGTCCCATATCAAGCGGCATACACTACAGATGCAGAGAGAAATGACGCAGTTTCTC
GTGACAAGCGTGCTCAGAGGGCGGTTTGGAATACTAATCTCCGTCTTCTAGAGGTGA
AGAAATCCGCCCTGGAGAAGAAGAAGACTGAGCTGGAGAGACGTCTGAAGGCGGAG
TTCAAGAAAGTGAATGAGCAGCAATCTGACTTGGGGGTTGGATATGCGAACTACCAG
TCTCCTTACCAGGCATAA

GSX7R sequence (2097bp)
GGGTTTGAGGCGACCGGCGACCGGCGTATTTATACGGCGGGGCCACAGCTAGCCGTTG
GGCCGGGCCGGCCGGGGGCAGTGGGCCAGTGAGGAGCGGGGCCTCTGCTTGGGGTG
GGCTGCGGAAAATGCTTCTCCTCGAACATTCGAAGCAAGCAGAAAAATCGGACAGGC
ACCTCTGACATACATATACCTATCCAAAGTTATATCTCTATCTAAAATCATATAACATGCA
CCTTTAACGACATTCAAAATGAATTTTCTCTTAAATTACTTATCTAATCTACAATCCGATC
ACACTGTTGTATTCGTTGTAATTAAATCTTTACAACAAGATGGCGCATGATTATATTCCG
ATTAAAAATAATTATATGTTTCAATCCATTCACACATTTTGTTTCATACGTGATAGGGACA
TGTTTCAATATGTGTCTTAAGCGTGTTTCATAAAATTCACAAAACTAACGGTTACTACTC
TCTCCTCTCTCTCTCTCTCAACCTCATGCATGCATGCATTTTAGCTAGTTTCAAAACGAT
TTTTCTCTTAAACTAATTATCCAATCTACGATCCGATCACACTGTTGTATTCTTTGTAATT
AATTTTTTACAACAAGATATCGCATGATTAAATTTTGATGAAAGAAAAACATATGTTTTA
ATCCATTAACATTGGTTGTTTCATATGTGATAGCCATATGTTTCAACGTGTATTTTCATTAT
GTTTCATAGACTATTCTCATTATGTTTCATAGACTTTTTAAATGTTTCAGTTGCTATTTTTT
GGTTTCACCCAATAAAACCTGGTGTTTCACTCATTAGTTAACTGAAACATTGGACTAAC
TGATTTTTTTTTGCATGCTACATACGTAGGTAGTGGAATATCAGACGTCCGACGCGGAG
TCGTCTCCGTGGGCTGCGCTAGCTTGCTTTGCACTCCTGTTCGCTCCCAGCCAGTTGCT
GCCCCTGCTATATCTGACTAACCACCGTGGAAATGCTCTGCGAGCTGTCACATCTTCGA
ATCGCTAAAGCACCTCCTCCCCTCCGCTCCTTCACTGCCATGTGGACCCATGCTCTCTC
ACAGCCACTGCCGCTGGAGGTTACCGATGACGCTCGCCGCCCGGGTTAGGGGGATAGG
ATTTCTGGAGGTTCCTGTCCTGGTGGCTCTAGAGGGAGGGCCGGGGGTGACCGGCAG
GACCGGCGGCAACGGCAGGCGCGCTGCCGCGGGCGATGCAGGCCAGCAATTGGGTCG
GTGATGGCGGCGGGGGCAGTGAATCCTAATAGGTTAGCGCGGGGGCGGGGGCTCCGG
CGGTGGCTACGGCCTGAGTCGGGAGCCGGGAGGGAATTCCATCGCCGCCGGAGCCAG
GAGGGAAGGCGGCGGGGGCTTGGGGTAGAGGGGTTAGTGTTGGGGGTGGAGACGGG
AGGGTGGCGGCGGAGGCGGGGGCTCTTGACGGTCGTTACCGACAAAGTTCGACCATC
AATTTAACTAAAATAATGGAGAATTAGCTATGCTTACAATGCATTTCATTTCCAAATAAT
GTCTATCTCCACTTGTACTTAGAAAATAACATGTTGCAGGAATACTTCACTTCTAAGCA
AGCTAGAGGATAAGGAGGGCCCACATGTCAGGTGTAACCGTCCGGAAACCGCCTTAAC

TGCCACAACCGCCTAAAGGACCCACCTGTCAGTGCCTGTACCGAAGCTGGAGGCCTG
CTGGGCCAGCGGGTTCGGCTGAACCCTGGCGGCGCCCAGACATGACGGCCCTCCATGT
GGACGCTCCAGAGTACTCCTCAATGACGGTTGCAAGGGCAAAACCGACTTTTCGTGTT
CCTACAACCGTTATAACTACCCTATAAAAGGAGGAGCTCATTCACTCTCTCAACACACT
CAAGCAAGCTCAATTCCTCTCATACTTTAGTATTAGTAGTATAGTGCTAAGAGGAGTGG
AAATAGAATAGTATTCAGAGTCCCTCGGAGTCTTCGGAAGAGCTTCGGTATGGCTCTAG
CAGCTCTTGTACTCTCTTTTGTAATACTTTCCGAATTAATGAAATACTCTTCTTTATATATC
ATCGGTATTCTGCTTAGTAATAAACATATGTAACC
