# Supplementary material for: Isolation and Functional Characterization of a Green-Tissue Promoter in Japonica Rice (Oryza sativa subsp. Japonica)
Source: Biology (Basel). 2022 Jul 22;11(8):1092. doi: 10.3390/biology11081092 (PMC9332004; doi:10.3390/biology11081092)
Supplement: Supplementary file 1 [file biology-11-01092-s001.zip › Table S1.pdf]

Table S1 *Cis*-elements related to green tissue specific expression in promoter sequence by PlantCARE

Cis-elements	Sequence	Position from transcription start site	Function/response
ACE	CTAACGTATT	-1273	involved in light responsiveness
Box 4	ATTAAT	+592，-2034	part of a conserved DNA module involved in light responsiveness
CAT-box	GCCACT	+1077	related to meristem expression
G-Box	CACGTT	-699，-1050	involved in light responsiveness
G-box	TACGTG	+399，+1051	involved in light responsiveness
GATA-motif	GATAGGA	+404，+1597，+1126	part of a light responsive element
GC-motif	CCCCG	-70，-1291，-1256，-1432，-1173，-1372，-1285	enhancer-like element involved in anoxic specific inducibility
I-box	GGATAAGGAG	-1598，+1599	part of a light responsive element
Sp1	GGGCGG	-1110，+1288	light responsive element
chs-Unit 1 m1	ACCTAACCCGG	-1114	part of a light responsive element
